# WHO SPECS 2030 – a global initiative to strengthen refractive error care

**Published:** 2024-05-15

**Authors:** Stuart Keel, Andreas Mueller

**Affiliations:** 1 Technical Officer: Sensory Functions, Disability and Rehabilitation Unit, World Health Organization, Geneva, Switzerland.; 2 Technical Advisor: Sensory Functions, Disability and Rehabilitation Unit, World Health Organization, Geneva, Switzerland.

**Coordinated global action is needed to improve access to refractive error services for everyone who needs it.**

**Figure:**
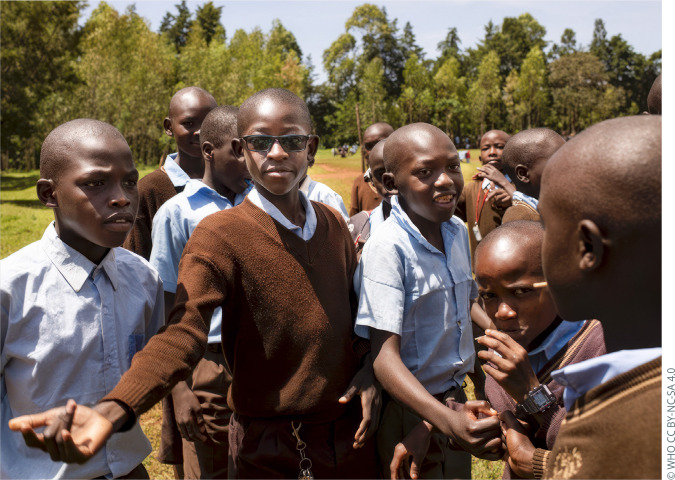
Spectacles improve access to education. kenya

Globally, it is estimated that only around one-third of people with vision impairment due to refractive error have access to a pair of spectacles that can effectively address their refractive error and allow them to see well.^1^ In recognition of the fact that uncorrected refractive error is the leading cause of near and distance vision impairment worldwide, and that spectacles are a very cost-effective intervention, a new global target for refractive error was endorsed at the World Health Assembly in 2021. Specifically, the global target is to increase the percentage of people with access to appropriate spectacles (known as effective coverage of refractive error, or eREC), by 40 percentage points by 2030.^2^ This means that, if the global coverage was 30% in 2020, the aim would be to achieve 70% coverage in 2030.

## What are the key challenges to achieving the global 2030 target for refractive error?

There are many challenges to achieving the 2030 global target for refractive error. First, uncorrected refractive error tends to be much greater in populations that already have inadequate access to health care.^3^ Second, refractive error and optical services are commonly only available in the private sector, and therefore costs pose a major barrier.^4^ Other challenges include the insufficient availability of qualified personnel to carry out refraction and dispense spectacles, limited government oversight and clinical regulation, scarce services outside of urban areas, and low awareness and acceptance of spectacles among members of the public.

## What is the WHO SPECS 2030 Initiative?

**Figure 1:**
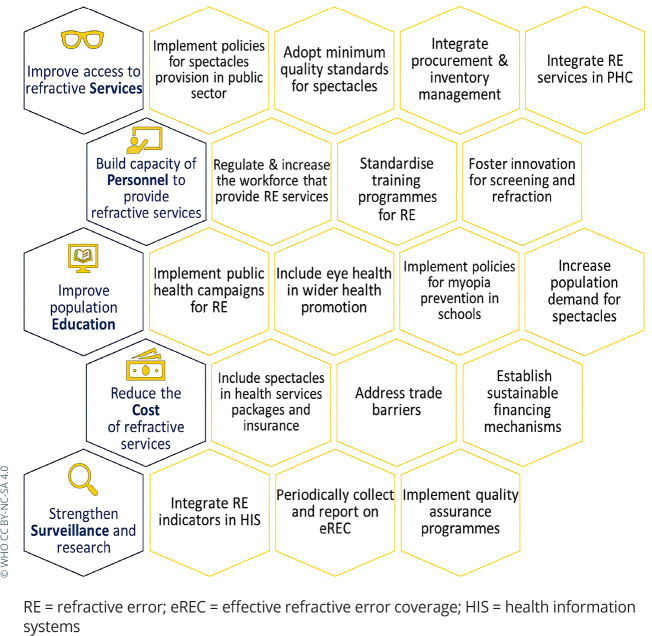
The five strategic pillars of the WHO SPECS 2030 Initiative (in blue) and the country-level desired outcomes (in yellow).

A comprehensive approach is needed to address these many long-standing challenges to increasing the coverage of refractive error services and – in particular – to strengthen provision in the government sector. The WHO SPECS 2030 Initiative calls for coordinated action amongst all stakeholders (public, private, non-profit, and philanthropy) across the following five pillars, in line with the SPECS acronym (Figure 1, in blue):

- **S:** Improve access to refractive **Services**
- **P:** Build capacity of **Personnel** to provide refractive services
- **E:** Improve population **Education**
- **C:** Reduce the **Cost** of refractive error services
- **S:** Strengthen **Surveillance** and research.

For each pillar, a set of country-level ‘desired outcomes’ have been defined by WHO, with input from the eye care sector,^5^ that would facilitate a sustainable increase in refractive error coverage (Figure 1, in yellow).

The WHO SPECS 2030 Initiative aims to support the achievement of this target in four key ways.

### 1. Developing technical guidance and tools

This area of work will include, but is not limited to, the development of the following:

- Guidance on legislative issues that impact on increasing spectacle coverage, for example, integration of refractive error services into primary health care
- Models for competency-based teams for refractive error services
- A costing tool to support country planning
- Tools to strengthen monitoring and surveillance.

### 2. Bringing together the key providers and supporters of eye care to collectively promote and advocate to governments

WHO has set up the WHO Global SPECS Network (www.who.int/initiatives/specs-2030/global-specs-network) to bring together intergovernmental organisations, non-governmental organisations, academic institutions, the private sector, and philanthropic foundations.

“Organisations and institutions can apply to become members of the Global SPECS Network.”

### 3. Motivating the private sector to make long-term contributions

WHO will convene a series of dialogues – the SPECS Private Sector Dialogues – with the optical, pharmaceutical, and technology industries, private sector service providers, and insurance companies. The dialogues will focus on how these groups can contribute to scaling up refractive error coverage, specifically targeting low- and intermediate-resource settings, in a way that will improve access and reduce the cost of refractive error services, especially in underserved populations.

### 4. Engaging with regions and countries

This may include WHO-led policy dialogues with governments to develop or strengthen refractive error services that are part of health systems, country-level workshops and training for health planners and health care providers, or capacity building and awareness raising within WHO regional and country offices.

**Figure:**
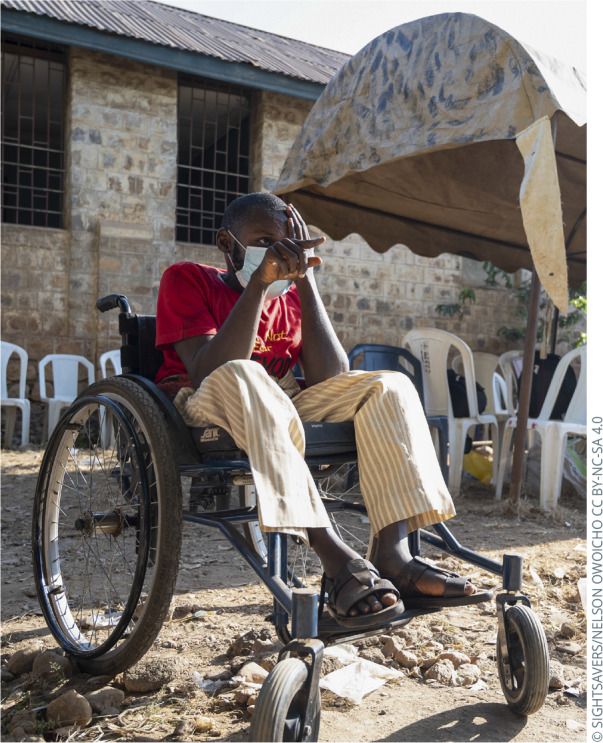
Refractive error services should be available to all, regardless of the type of health condition or impairment an individual may have. nigeria

## How to contribute to the WHO SPECS 2030 Initiative

**Individuals** who are involved in the provision and/or coordination of eye care are encouraged to advocate for the need to strengthen the provision of refractive services at all levels of the health system. To support this, a series of specific technical resources are available on the WHO SPECS 2030 website (www.who.int/initiatives/specs-2030). Additional resources will be added throughout 2024 and 2025.

**Organisations and institutions** can apply to become members of the Global SPECS Network. Further information regarding the eligibility criteria and how to apply can be found on the Global SPECS Network webpage (www.who.int/initiatives/specs-2030/global-specs-network).

Finally, **private sector representatives** are encouraged to join the SPECS Private Sector Dialogues already mentioned, for which registration will open in mid-2024.

For more information, please contact the WHO Vision and Eye Care Programme (vision@who.int).

**Figure:**
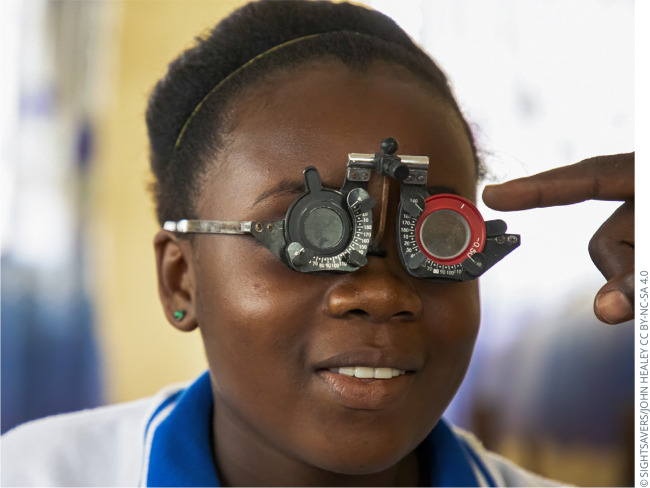
School eye screening programmes cannot function without the support of adequate refractive error services. liberia

## References

[1] World Health Organization. Report of the 2030 targets on effective coverage of eye care [Internet]. Geneva: World Health Organization; 2022 (https://apps.who. int/iris/handle/ 10665/363158, accessed 20 December 2023).

[2] Resolution WHA74(12). Integrated people-centred eye care, including preventable vision impairment and blindness. In: Seventy-fourth World Health Assembly, Geneva, 24–31 May 2021. Geneva: World Health Organization; 2021

[3] WHO. World report on vision. Geneva: World Health Organization; 2019.

[4] WHO. Package of eye care interventions. Geneva: World Health Organization; 2022.

[5] WHO. Global Refractive Error Expert Consultation, WHO Headquarters, Geneva, Switzerland, Meeting Report; 2023.

